# The effect of amniotic membrane application on post-cesarean wound healing and cosmetic outcomes

**DOI:** 10.1038/s41598-025-23623-6

**Published:** 2026-06-30

**Authors:** Yusuf Başkıran, Kazım Uçkan, İzzet Çeleğen, Fatma Başak Tanoğlu

**Affiliations:** 1 Faculty of Medicine, Department of Obstetrics and Gynecology, Istinye University, Istanbul, Turkey; 2 Faculty of Medicine, Department of Obstetrics and Gynecology, Van Yuzuncu Yil University, Van, Turkey; 3 Faculty of Medicine Department of Public Health, Yuzuncu Yil University, Van, Turkey; 4 Acibadem Healthcare Group, Department of Obstetrics and Gynecology, Istanbul, Turkey

**Keywords:** Amniotic membrane, Cesarean section, Wound healing, Surgical site infection, Postoperative pain, Outcomes research, Randomized controlled trials

## Abstract

Post-cesarean wound complications—such as surgical site infection (SSI), dehiscence, and hypertrophic scarring—remain a challenge in obstetric surgery. We evaluated whether intraoperative autologous amniotic membrane application improves postoperative outcomes. In a single-blind, randomized controlled trial, 372 women undergoing elective cesarean section were allocated to an amniotic membrane group (*n* = 174) or control group (*n* = 198). SSI incidence was lower with amniotic membrane (4.3% vs. 10.8%; *p* < 0.05), and wound dehiscence was reduced (2.1% vs. 6.4%; *p* < 0.05). Postoperative pain scores (Visual Analog Scale) were significantly lower in the amniotic membrane group on postoperative day 1 (3.38 ± 1.36 vs. 4.05 ± 1.53; *p* = 0.01) and day 2 (1.25 ± 0.71 vs. 2.01 ± 0.98; *p* = 0.001). Clinician-rated cosmetic appearance (Modified Hollander Wound Evaluation Scale) did not differ between groups (*p* = 0.863), whereas patient-reported satisfaction was higher with amniotic membrane (*p* = 0.001). No predefined adverse events were observed. These findings suggest that amniotic membrane application may reduce wound-related complications and postoperative pain and improve patient satisfaction after cesarean delivery; confirmatory studies in broader populations are warranted.

**Supplementary Information:**

The online version contains supplementary material available at 10.1038/s41598-025-23623-6.

## Introduction

Cesarean section (CS) is one of the most commonly performed surgical procedures worldwide^1^. Despite its prevalence, complications such as surgical site infection (SSI), hematoma, wound dehiscence, and keloid formation significantly impact patient quality of life and increase the economic burden on healthcare systems^2^. Local factors, such as tissue oxygenation, necrotic tissue, and suboptimal surgical techniques, along with systemic factors like advanced age, diabetes mellitus, and sepsis, further complicate the wound healing process^3,4^.

Wound healing is a complex mechanism involving cellular, physiological, and biochemical processes, occurring in three main phases: inflammation, proliferation, and maturation^5^. Factors disrupting this mechanism often lead to complications such as hypertrophic scar or keloid formation, which not only present cosmetic concerns but also cause physical and psychological distress, including pain, itching, and emotional stress^6^. Although various treatments, such as intralesional steroids, surgical excision, and laser therapies, are available, their success rates vary and often require multiple interventions^7^.

The amniotic membrane, the innermost layer of the placenta, has emerged as a promising material in wound healing due to its unique properties, including anti-inflammatory, antimicrobial, angiogenic, and epithelialization-promoting effects^8^. It has been widely used in treating chronic wounds, corneal injuries, and tissue adhesion prevention^9^. Recent studies also suggest its potential application in advanced regenerative therapies, such as spinal cord injury and neurodegenerative diseases^10^.

This study is the first to evaluate the intraoperative application of the amniotic membrane in a low-risk cesarean population, aiming to make a unique contribution to the literature by optimizing wound healing and preventing postoperative complications.

## Materials and methods

This single-blind, prospective randomized controlled trial was conducted using a parallel group design, with participants randomized in a 1:1 ratio (block size = 4). The study was carried out between June 2022 and June 2023 at the Department of Obstetrics and Gynecology, Van Regional Training and Research Hospital. Although block randomization was applied, logistical factors during recruitment resulted in a slight imbalance between groups (174 vs. 198 participants). To address this, all outcome analyses were re-checked using logistic regression models adjusted for baseline characteristics (age, BMI, parity, incision length, subcutaneous tissue thickness, and wound closure time). These adjusted analyses yielded results consistent with the unadjusted findings, confirming that the imbalance did not materially affect the study conclusions. The surgical team was aware of the intervention, but outcome assessors remained blinded to minimize bias.

### Participants and eligibility criteria

Eligible participants were women aged 18–40 years with term pregnancies undergoing their first elective cesarean section (CS) under spinal anesthesia using the Pfannenstiel incision method. Only elective cases were included, and patients with any signs of infection during delivery were excluded to minimize contamination risk. Additional exclusion criteria were: previous abdominal surgery, chronic diseases (e.g., diabetes, hypertension, vascular or pulmonary conditions, collagen tissue disorders), and maternal or fetal infection at the time of surgery.

All participants provided written informed consent, and the study was approved by the Ethics Committee of Van Regional Training and Research Hospital (approval number: 2022/22 − 05). The trial was retrospectively registered in ClinicalTrials.gov (NCT06770946; registration date: 31/12/2024). The retrospective registration was due to administrative delays; however, all primary and secondary outcomes and the statistical analysis plan were pre-specified before data collection and remained unchanged throughout the study.

All procedures were conducted in accordance with institutional guidelines and the ethical principles of the Declaration of Helsinki. The process of study selection is presented in Supplementary Fig. 1.

## Intervention

The intervention group received intraoperative autologous amniotic membrane application, while the control group underwent standard cesarean delivery without this procedure. The membrane was harvested from the fetal side of the placenta immediately after delivery, trimmed to match the incision size, and placed directly over the surgical site prior to skin closure. It was kept in place for 24 h and removed during the first postoperative dressing change. Both groups received identical standard postoperative wound care.

**Fig. 1:**
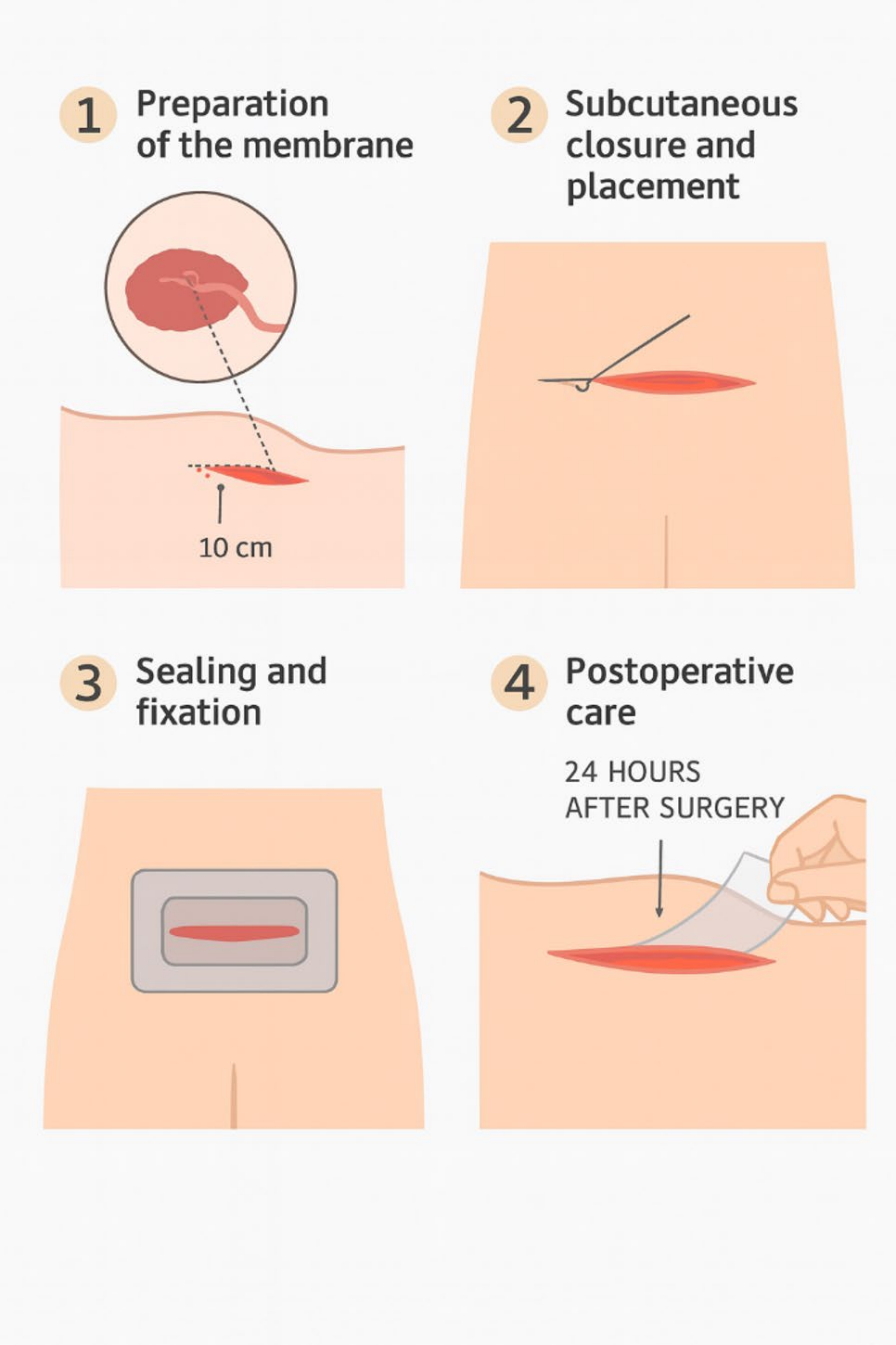
Schematic representation of the autologous chorioamniotic membrane application technique used in the intervention group.

A stepwise schematic representation of the harvesting, preparation, placement, fixation, and postoperative management of the amniotic membrane is provided in Fig. 1.

(1) *Preparation of the membrane*: The chorioamniotic layer is harvested from the placental region near the umbilical cord and thoroughly cleansed to remove blood residues. A segment approximately 10 cm in length is trimmed to match the cesarean incision size.

(2) *Subcutaneous closure and placement*: Following subcutaneous closure with 3.0 Rapid Vicryl, the prepared membrane is placed directly over the skin incision, ensuring full coverage and contact.

(3) *Sealing and fixation*: A transparent, waterproof dressing (e.g., Tegaderm) is applied to secure the membrane in place and protect it from external exposure.

(4) *Postoperative care*: After 24 h, the dressing is removed, and the membrane is inspected for adherence and hydration. Follow-up is initiated to monitor integration and healing progression.

## Outcomes and follow-up

### Primary outcomes

Surgical site infection (SSI) (diagnosed based on erythema, induration, tenderness, and purulent discharge), hematoma, seroma (defined as serosanguineous discharge without signs of infection), wound dehiscence, and scar or keloid formation.

### Secondary outcomes

Postoperative pain (assessed using the Visual Analog Scale, VAS), cosmetic outcomes (evaluated using the Modified Hollander Wound Evaluation Scale)^11^, and patient satisfaction. Outcomes were assessed on postoperative days 1, 2, 7, and 40, as well as at 6 months^12^.

### Safety outcomes

Adverse events were predefined as allergic reactions, excessive bleeding, wound necrosis, systemic infection, need for reoperation, or unexpected hospital readmission within 6 months.

## Randomization and blinding

Participants were randomized using a computer-based block randomization program with a block size of 4 to ensure balanced group allocation. Despite this approach, a slight imbalance was observed between groups (174 vs. 198 participants). To address this, outcome analyses were re-checked using logistic regression models adjusted for baseline variables (age, BMI, parity, incision length, subcutaneous tissue thickness, and wound closure time), and the results remained consistent with the unadjusted analyses.

The surgical team was aware of the intervention, but all outcome assessors remained blinded to minimize bias. Amniotic membranes were prepared and coded separately to maintain blinding. To ensure effective blinding, all postoperative outcome assessments—including pain scores, wound evaluation, and patient satisfaction—were performed by independent clinicians who were not involved in the surgical procedures. These assessors were provided with anonymized patient identifiers and were unaware of group allocation. Subjective outcomes were recorded using standardized interview protocols to reduce potential observer bias.

## Sample size calculation

The sample size was calculated based on reported SSI prevalence rates (5%–12%), aiming to detect a reduction in SSI rates from 10% (control group) to 4% (intervention group) with 80% power (β = 0.20) and an alpha level of 0.05. To account for potential dropouts, an additional 10% of participants were planned for recruitment, yielding a target sample size of approximately 372 women. In practice, 372 participants were enrolled, but due to logistical factors during recruitment, group allocation resulted in 174 participants in the intervention group and 198 in the control group.

### Statistical analysis

All statistical analyses were conducted using IBM SPSS Statistics 20. Continuous variables were assessed for normality using the Shapiro–Wilk test and reported as mean ± standard deviation (SD) for normally distributed data or median (minimum–maximum) for non-normally distributed data. Categorical variables were expressed as numbers and percentages and compared using Fisher’s exact test. VAS scores, cosmetic evaluation, and patient satisfaction were compared using the independent t-test or Mann–Whitney U test depending on data distribution.

To account for the slight imbalance in group sizes (174 vs. 198), all primary outcome analyses were additionally re-checked using logistic regression models adjusted for baseline variables (age, BMI, parity, incision length, subcutaneous tissue thickness, and wound closure time). Adjusted results were consistent with unadjusted findings, confirming the robustness of the analyses.

A p-value of < 0.05 was considered statistically significant.

## Results

A total of 372 patients were randomized into two groups: 174 participants in the amniotic membrane group and 198 in the control group. No dropouts or exclusions occurred post-randomization. Recruitment took place between June and December 2022, and follow-up assessments were completed by June 2023. Despite block randomization in a 1:1 ratio, a slight imbalance between the groups was observed due to practical challenges in patient enrollment and allocation, though statistical adjustments were made accordingly. Baseline demographic and clinical characteristics, including age, BMI, gravida, parity, incision length, subcutaneous tissue thickness, and wound closure time, were comparable between the two groups, with no statistically significant differences (Table 1).

**Table 1:** Medical information and incision characteristics of the study groups.

	Amniotic (*n* = 174) Mean ± SD	Control (*n* = 198) (min-max)	Amniotic (*n* = 174) Mean ± SD	Control (*n* = 198) (min-max)	*p*
Age	25.5 ± 3.9	(19–32)	25.6 ± 4.4	(19–33)	0.932
Bmi	29.3 ± 4.1	(22–38)	28.5 ± 4.1	(22–38)	0.314
Incision length(mm)	106.5 ± 10.1	(95–130)	108.0 ± 10.1	(81–130)	0.418
Subcutaneous thickness	25.5 ± 10.4	(10–54)	24.9 ± 8.9	(10–46)	0.698
Wound closure time(sec)	247.6 ± 49.3	(194–364)	244.0 ± 40.1	(196–370)	0.659
Gravida	2.2 ± 0.7	(1–4)	2.1 ± 0.8	(1–4)	0.524
Parity	1.0 ± 0.7	(0–3)	1.0 ± 0.7	(0–3)	0.592

Abbreviations: SD; Standard deviation, BMI; body mass index. Findings are presented as mean±standard deviation (minimum−maximum), *p *< 0.05 is statistically significant.

## Primary outcomes

**Fig. 2:**
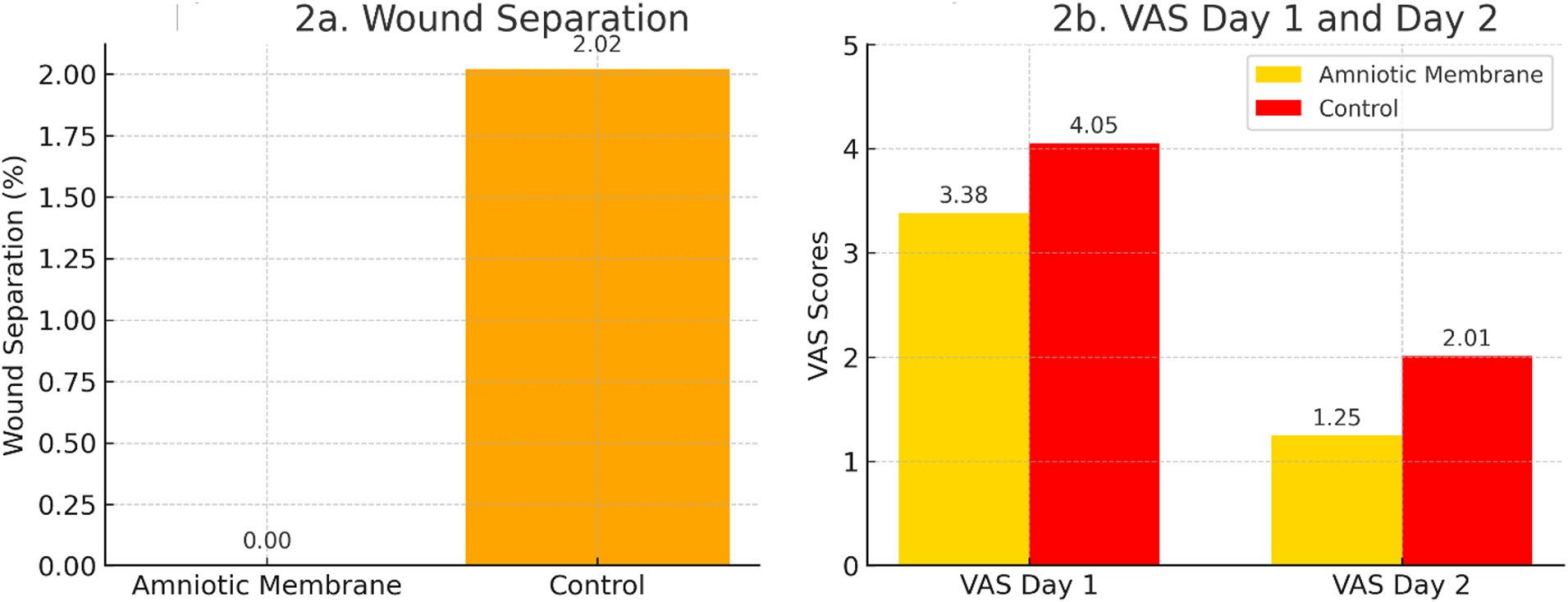
**a** Wound separation (%) following cesarean delivery with or without intraoperative amniotic membrane application.**b** Postoperative pain scores (VAS) on days 1 and 2 after cesarean delivery with or without intraoperative amniotic membrane application.

The incidence of surgical site infection (SSI) was lower in the amniotic membrane group (4.3%) compared to the control group (10.8%) (*p* < 0.05). Wound dehiscence was observed in 2.1% of the amniotic membrane group and 6.4% of the control group (*p* < 0.05). These differences in wound separation are illustrated in Fig. 2a. Hematoma was reported in two patients in the control group on postoperative day 2, but no cases were detected in the amniotic membrane group. Similarly, seroma was observed in one patient from the control group on postoperative day 7, whereas none occurred in the amniotic membrane group. By postoperative day 40 and at the 6-month follow-up, scar and keloid formation were detected in four patients from the control group but were absent in the amniotic membrane group (*p* < 0.05) (Table 2).

**Table 2:** Incidence of wound complications in the study Groups*.

		Postop. 2nd day		Postop. 7th day		Postop. 40th day		Postop. 6th month	
		Group 1	Group 2	Group 1	Group 2	Group 1	Group 2	Group 1	Group 2
Hematoma	Yes	0	2	0	0	0	0	0	0
Seroma	Yes	0	0	0	1	0	0	0	0
Infection	Yes	0	0	1	6**	0	0	0	0
Wound opening	Yes	0	0	0	4**	0	0	0	0
Scar keloid formation	Yes	0	0	0	0	0	4**	0	4**

*The numbers in the table indicate the number of patients.**p < 0.05 is statistically significant (Fisher exact test).

### Secondary outcomes

Postoperative pain scores, measured using the Visual Analog Scale (VAS), were significantly lower in the amniotic membrane group on postoperative day 1 (3.38 ± 1.36 vs. 4.05 ± 1.53; *p* = 0.01) and day 2 (1.25 ± 0.71 vs. 2.01 ± 0.98; *p* = 0.001). These results are presented in Fig. 2b. However, cosmetic healing, assessed using the Modified Hollander Wound Evaluation Scale, did not differ significantly between the groups (*p* = 0.863). Patient satisfaction levels were significantly higher in the amniotic membrane group (*p* = 0.001) (Table 3).

**Table 3:** Pain scores, cosmetic results and satisfaction levels of the groups with and without amniotic membrane application.

	Amniotic (*n* = 174)	Control (*n* = 198)	P**
VAS score; day 1	3.38 ± 1.36	4.05 ± 1.53	**0.01**
VAS score; day 2	1.25 ± 0.71	2.01 ± 0.98	**0.001**
Modified Hollander; day 40	5.86	5.85	0.863
Satisfaction survey; day 40	4.6/5	3.2/5	**0.001**

Bold values indicate statistically significant differences between groups (p 0.05)

Findings are presented as mean±standard deviation.**p < 0.05 is statistically significant

### Safety and adverse events

No predefined adverse events (allergic reactions, excessive bleeding, wound necrosis, systemic infection, need for reoperation, or unexpected hospital readmission) were observed in either group. Minor postoperative complaints (e.g., mild erythema, transient discomfort) occurred but did not require intervention.

The figure visually summarizes the differences between the intervention and control groups, showing reduced wound separation and lower postoperative pain scores (VAS) on days 1 and 2 in the amniotic membrane group.

## Discussion

Cesarean section (CS) remains one of the most frequently performed abdominal surgeries worldwide, yet postoperative complications such as surgical site infection (SSI), hematoma, wound dehiscence, and keloid formation continue to challenge both patient outcomes and healthcare costs. Given the nature of surgical interventions, achieving complete blinding in randomized trials involving intraoperative applications is inherently difficult. Identifying factors that influence wound healing and implementing strategies that are not only effective but also practical and safe remain critical objectives for obstetric surgeons. Although CS is generally considered a safe elective procedure, SSI rates vary depending on factors such as subcutaneous tissue thickness, surgical technique, and perioperative care. While interventions such as negative pressure wound therapy (NPWT) and antimicrobial sutures have been investigated to reduce wound complications, their cost and applicability limit their widespread use, particularly in low-risk populations. Consequently, current research continues to focus on finding affordable and feasible approaches that optimize surgical outcomes while maintaining patient safety.

Various strategies have been investigated to improve wound healing outcomes and minimize postoperative complications after surgery. For instance, Kumutnart et al. demonstrated that a 12% topical onion extract gel, applied three times daily for 12 weeks, significantly improved the cosmetic appearance of surgical scars compared with placebo^13^. Similarly, a meta-analysis of prophylactic negative pressure wound therapy (NPWT) reported a 58% reduction in infection rates, decreasing from 12.5% to 5.2% relative to standard wound dressings^14^. However, the cost and limited accessibility of NPWT restrict its routine use, making it primarily applicable to high-risk patients, such as those with diabetes, immunosuppression, or a body mass index (BMI) ≥ 30 kg/m²^15^. Given that postoperative infection rates vary depending on patient characteristics and perioperative conditions, further research is warranted to identify the most effective and cost-efficient strategies for SSI prevention, particularly in elective CS cases. While NPWT remains a well-established intervention in high-risk surgical patients, alternative biologically active dressings—such as the amniotic membrane—may provide a safe and cost-effective option for optimizing wound healing.

Current wound healing strategies have notable limitations, including the need for prolonged monitoring, challenges with patient adherence, and an increased workload for healthcare providers. Moreover, their costs often approximate or even exceed the expenses of managing postoperative complications, thereby restricting accessibility—particularly for low-risk populations undergoing elective cesarean sections. Given the reported variability in infection rates after elective surgery, there remains a pressing need to identify interventions that are cost-effective, safe, and broadly applicable. This underscores the importance of innovative, practical solutions that can be seamlessly integrated into routine clinical practice to optimize wound healing outcomes while minimizing the burden on healthcare systems.

The therapeutic potential of the amniotic membrane was first recognized in 1910, when Davis reported its use in skin grafts^16^. Since then, its applications have expanded across multiple medical fields, with growing evidence supporting its role in wound healing. Schmiedova et al. reviewed studies published between 2000 and 2020 and emphasized the membrane’s beneficial effects in chronic wound management^8^. Its high hyaluronic acid content helps limit excessive fibrosis, supports epithelialization, and reduces scar formation through the release of growth factors such as epidermal growth factor (EGF), keratinocyte growth factor (KGF), and hepatocyte growth factor (HGF)^16^. In addition, its angiogenic properties—mediated by basic fibroblast growth factor (bFGF) and transforming growth factor-beta (TGF-β)—facilitate tissue regeneration. The membrane also exhibits anti-inflammatory activity via interleukin-10 (IL-10) and thrombospondin-1, which suppress excessive tissue reactions. Importantly, it lacks major histocompatibility complex (MHC) antigens (HLA-A, B, C), thereby minimizing the risk of immune rejection. Collectively, these biological features position the amniotic membrane as a promising candidate for clinical use; however, its role in elective surgical settings, such as cesarean delivery, still requires further investigation to establish its safety and effectiveness.

Mao et al. demonstrated that the amniotic membrane possesses antibacterial properties, inhibiting the growth of Pseudomonas aeruginosa, Staphylococcus aureus, and methicillin-resistant S. aureus (MRSA), suggesting a potential role in reducing wound infections^17^. These antimicrobial effects have prompted its investigation across multiple medical fields, including chronic wound management and the prevention of postoperative tissue adhesions^18–20^. In 2020, Razazadeh et al. proposed the intraoperative application of autologous amniotic membranes during cesarean delivery to promote wound healing and highlighted its cost-effectiveness^15^. Similarly, Fan et al. demonstrated in a rat uterine scar model that human amniotic epithelial cell transplantation facilitated tissue repair and collagen degradation^21^. Despite these promising results, however, the direct use of the amniotic membrane in elective cesarean incisions has not been extensively studied. Given the unique considerations in obstetric surgery, including sterility and infection control, further high-quality clinical trials are required to confirm the safety, efficacy, and feasibility of this intervention in routine practice.

Our single-blind, prospective randomized controlled trial represents one of the first investigations to evaluate the intraoperative application of autologous amniotic membrane to cesarean incision sites. The findings demonstrated significant reductions in postoperative complications, particularly wound infection and dehiscence. Patients in the amniotic membrane group also reported lower postoperative pain scores and higher overall satisfaction. Although cosmetic healing assessed by the Modified Hollander Wound Evaluation Scale did not differ significantly between groups, patient satisfaction was markedly higher in the intervention group. This apparent discrepancy may be explained by the subjective nature of satisfaction, which is often influenced by factors beyond objective wound appearance—such as reduced pain, greater comfort during recovery, and psychological reassurance. Previous studies have similarly reported divergences between patient-reported satisfaction and clinician-rated cosmetic outcomes in postoperative settings. Importantly, no predefined adverse events (including allergic reactions, excessive bleeding, wound necrosis, systemic infection, reoperation, or hospital readmission) were observed, underscoring the favorable safety profile of this intervention. While these findings are encouraging, the study was limited to low-risk cesarean patients, and additional research is warranted to determine its applicability in higher-risk populations.

Our findings highlight the potential of this low-cost and easy-to-apply intervention as a practical strategy to improve surgical outcomes and enhance patient satisfaction. Nevertheless, wound healing is a complex and multifactorial process, and complications may still occur even in low-risk populations, underscoring the unpredictable nature of tissue repair. The autologous, intraoperative applicability of the amniotic membrane, combined with its cost-effectiveness, further supports its potential for broader clinical use. However, considering the critical importance of sterility and infection control in obstetric surgery, further well-designed studies with stringent protocols are required to confirm its safety and efficacy across different clinical settings.

This study has several limitations. First, due to the nature of the intervention, complete double-blinding was not feasible, as the surgeons were necessarily aware of the treatment allocation. Second, although block randomization with a block size of four was applied, a slight imbalance between groups occurred, most likely due to logistical factors during enrollment. Third, the retrospective registration of the trial, completed in December 2024, represents a limitation in terms of transparency and strict adherence to prospective trial guidelines. In addition, the study sample consisted exclusively of low-risk patients undergoing elective cesarean sections, which restricts the generalizability of the findings to high-risk populations or emergency procedures. Moreover, while the study was adequately powered to detect differences in the primary outcomes, it may have been underpowered to assess rare complications or predefined adverse events. Patient-reported outcomes, such as cosmetic satisfaction and pain, are also inherently subjective and influenced by individual perceptions. Finally, the follow-up period was limited to six months, which may have overlooked long-term outcomes such as delayed scar formation or chronic pain. Future studies involving more diverse populations, larger cohorts, and extended follow-up are needed to validate and expand upon these results.

## Conclusion

The application of the amniotic membrane during cesarean delivery demonstrates potential as a prophylactic strategy for improving wound healing and preventing postoperative complications. This intervention was associated with a reduced incidence of surgical site infections, wound dehiscence, and scar formation, while also alleviating postoperative pain and improving patient-reported satisfaction. Importantly, no predefined adverse events were observed, further supporting the favorable safety profile of this approach. Nevertheless, given the nature of elective cesarean surgeries, strict sterility protocols are required when considering amniotic membrane application to minimize potential risks. While these findings suggest that this technique could be a practical and cost-effective addition to surgical wound care, further research in high-risk populations and emergency settings is essential to determine its broader applicability.

This study represents one of the first randomized controlled trials to evaluate the efficacy of amniotic membrane application specifically on cesarean incisions in a low-risk population. Although these results are encouraging, additional research with larger and more diverse cohorts, including patients undergoing emergency cesarean sections or presenting with comorbidities, is needed. Moreover, future studies should investigate long-term outcomes, such as delayed scar formation and chronic pain, to provide a more comprehensive understanding of the therapeutic potential and broader clinical implications of amniotic membrane use in surgical practice.

## Supplementary Information

Below is the link to the electronic supplementary material.

Supplementary Material 1

Supplementary Material 2

## Data Availability

The data used and analyzed during this research are available from the corresponding author upon reasonable request.
