# Supplementary figures and images for: The effect of amniotic membrane application on post-cesarean wound healing and cosmetic outcomes
Source: Sci Rep. 2026 Jun 30;15:37532. doi: 10.1038/s41598-025-23623-6 (PMC13315581; doi:10.1038/s41598-025-23623-6)

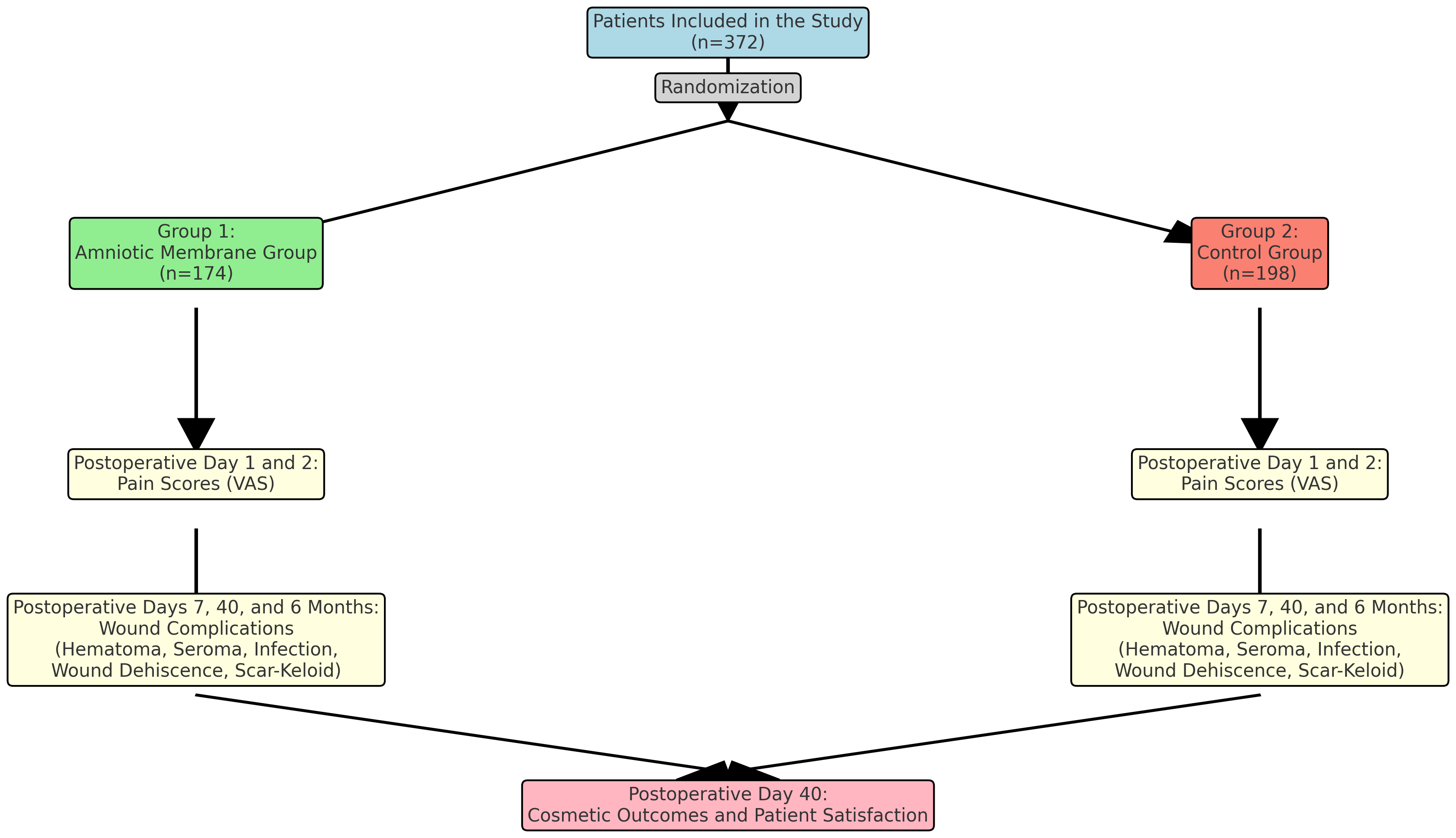

Supplement: Supplementary file 1 — Supplementary Material 1 [file 41598_2025_23623_MOESM1_ESM.png]
