# Supplementary material for: The effect of amniotic membrane application on post-cesarean wound healing and cosmetic outcomes
Source: Sci Rep. 2026 Jun 30;15:37532. doi: 10.1038/s41598-025-23623-6 (PMC13315581; doi:10.1038/s41598-025-23623-6)
Supplement: Supplementary file 2 — Supplementary Material 2 [file 41598_2025_23623_MOESM2_ESM.docx]

**Supplementary Figure Legends**

Supplementary Figure 1. Flowchart of the study design and follow-up assessments.

Legend: Diagram showing patient randomization (n=372) into amniotic membrane group (n=174) and control group (n=198). Postoperative pain scores were assessed on days 1 and 2; wound complications on days 7, 40, and 6 months; and cosmetic outcomes and patient satisfaction on day 40.
